# Rush Medical College

**Published:** 1869-01-15

**Authors:** 


***Rush Medical College***

Puts forth its annual announcement of the Spring Course of Instruction for 1869 in the present number of THE JOURNAL.

Every student of medicine is urged to consider the advantages to him of remaining in the city during the spring and summer, when the best opportunities are afforded for successful study. The hospitals and dispensaries are then overrunning with material illustrative of disease, and the student may have these facilities at a comparatively trivial expense.

He needs access to libraries and reading rooms; and, above all, he needs to become familiar with earnest men in the profession, that his character may be stamped by the example of their industry and habits of attention. He needs a text book, daily and hourly illustrated in the street; the class-room, the laboratory; the *clinique*, the hospital ward, and the dead-house, and the active rivalry of asssociates—a sure road to excellence.

The design of the course is that it may supply the want of a numerous body of students who must pass the time more or less unprofitably while removed from the best appliances during a long vacation. The majority of medical students in the North-west do not need to be reminded of the value of time or money. A year given to close observation under the tutelage of the college will insure practical ability with the prospect of immediate reward. The College offers to become Preceptor as well as *Alma Mater* that the highest luster may be given its *Alumni*.

